# Trends in Suicidology: Personality as an Endophenotype for Molecular Genetic Investigations

**DOI:** 10.1371/journal.pmed.0030107

**Published:** 2006-05-09

**Authors:** Jonathan B Savitz, Cinda-Lee Cupido, Rajkumar S Ramesar

## Abstract

In studying the genetics of suicide, should personality be used as an endophenotype (an intermediate trait lying somewhere on the developmental pathway from genes to phenotype)?

Psychopathology, especially depression, is the most important risk factor for suicidal behaviour [ 1, 2] with between 25% and 40% of depressed patients attempting suicide [ 3] and about 3.4% completing suicide eventually [ 4]. Given recent figures suggesting that the lifetime prevalence of a major depressive episode among the US population is 32.6–35.1 million [ 5], it is no surprise that suicide ranks among the top ten causes of death in many other countries [ 6].

Understanding the aetiology of a significant public health issue such as suicide is important but difficult because of its complex and multifactorial origins. Although most suicidal behaviour occurs within the context of a mood disorder, most depressed individuals never attempt suicide. Furthermore, no linear relationship between the severity of the depressive episode and the likelihood of suicide has been forthcoming [ 7], highlighting the importance of other factors in addition to psychiatric illness. These factors include substance abuse or alcoholism, a head injury, a dysfunctional family or childhood abuse [ 8], high rates of gun ownership [ 9], smoking [ 10], socioeconomic adversity [ 11], and personality factors [ 12].

Genetic factors may also be very important [ 13, 14]. Marusic and Farmer [ 15] argue that the variation in the suicide rate across European countries (7–43 per 100,000 inhabitants per year) cannot be explained by sociocultural factors alone and is probably due to shared genetic vulnerability. A case in point is the high suicide rate in Hungary and Finland, two populations with a common genetic origin but with divergent cultural and political trajectories [ 15]. [Fig pmed-0030107-g001]

**Figure pmed-0030107-g001:**
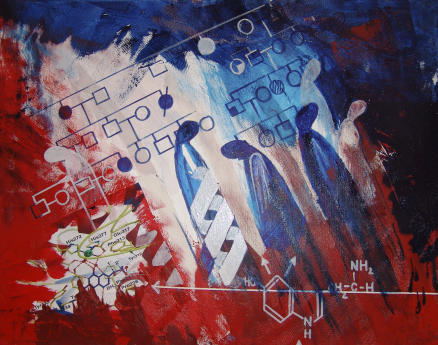
Colours of Depression (Illustration: Raj Ramesar)

At the family level, the risk of suicide is higher in individuals with a family history of suicide [ 16, 17], and the suicide rate of adolescents is highly correlated with the suicide rate among their relatives [ 18]. Even studies that have controlled for levels of psychopathology have shown that relatives of suicide completers and attempters are at an increased risk for suicidal behaviour [ 19, 20]. Twin studies indicate that this familial clustering of suicidal behaviour has a partly genetic basis with heritability estimates of 17%–55% for suicidal behaviour [ 21, 22] and 20% for suicide [ 23] reported. The only adoption study we are aware of suggested that as far as suicidal behaviour is concerned, adoptees resemble their biological parents more than the adoptive family [ 24].

These data have catalysed the search for genes that predispose to suicidal behaviour, with more than 100 studies now published [ 25]. Post-mortem studies of people who committed suicide have led to the general consensus that a disturbance of the serotonergic system is associated with suicidal behaviour. Therefore, it is no surprise that most of the genes implicated in suicidal behaviour—the serotonin transporter (SERT), tryptophan hydroxylase (TPH), monoamine oxidase A (MAO-A), and the serotonin receptors, 5-HTR1A, 5-HTR2A, and 5-HTR1B—modulate central serotonergic function (see Table 1). As is the case with most complex traits, however, success tends to plateau at a point where good candidate genes are identified but conclusive causal inferences remain elusive because of replication failures.

**Table 1:**
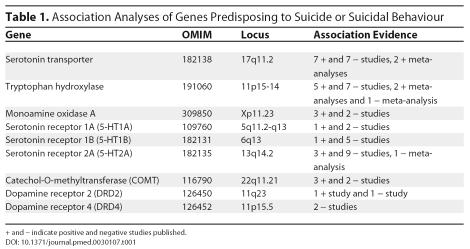
Association Analyses of Genes Predisposing to Suicide or Suicidal Behaviour

The unequivocal identification of genes related to psychiatric disorder is retarded by a complex interplay of latent environmental influences or gene–environment interactions; genetically and phenotypically heterogeneous samples; the possible effects of numerous loci of small effect size; and the difficulty of adequately correcting for multiple testing. Faced with these frustrations, the use of endophenotypes as an aid to molecular genetic investigations has become almost de rigueur. In the case of suicide, a number of researchers have advocated the use of personality traits as endophenotypes [ 12, 26].

## What Is an Endophenotype?

An endophenotype is an intermediate trait that lies somewhere on the developmental pathway from genes to phenotype [ 27]. If suicide is the phenotype of interest—the final product of different genetic and environmental factors—then the endophenotype is a more elementary trait that is tightly correlated with suicide. The genetic architecture of the endophenotype is assumed to echo its relative phenotypic simplicity, presenting a more tractable target for geneticists. An understanding of the molecular basis of the endophenotype should theoretically be the first step towards the larger prize: uncovering the molecular basis of the phenotype itself.

Gottesman and Gould [ 27] assert that for a biological marker to be classified as a bona fide endophenotype, it must meet the following conditions: 1) be associated with the illness in the relevant population; 2) be largely state-independent, manifesting in the individual during both periods of health and illness; 3) be heritable; 4) within families, the endophenotype and illness should co-segregate; 5) if found in affected individuals should also be found in nonaffected family members at a higher rate than in the general population.

In the following sections we use Gottesman and Gould's [ 27] five criteria above to evaluate the latest trend in suicide research: the merits of using an endophenotype such as personality to identify suicide susceptibility genes.

## Are Specific Personality Traits Associated with Suicide Behaviour?

Four main constellations of personality characteristics are associated with suicidal behaviour: impulsivity, hostility–aggression, introversion, and anxiety-neuroticism.

A rich body of data details the association between suicide attempts and impulsivity. Impulsivity has been shown to be a risk factor for suicidal behaviour in a variety of adults [ 7, 8, 28–30] and adolescents [ 11, 31, 32] with psychiatric illness, in a forensic psychiatric population [ 33], and in the general population [ 34].

Freud regarded suicide as an aggressive act, a view borne out to some extent by modern data. Simon et al. [ 35] found that involvement in physical fights was associated with violent suicide attempts, and, according to Appleby et al. [ 36], people who committed suicide were more likely to have been arrested in the previous six months. Cavanagh et al. [ 37] confirmed that a criminal record is a risk factor for subsequent suicide. Fulwiler et al. [ 38] detected a higher rate of suicide attempts in a group of violent compared with nonviolent patients with psychiatric illness, and in South Africa conduct disorder was found to be the most frequently made diagnosis in suicidal adolescents [ 39].

Negative affect or anxiety is most commonly measured in the form of neuroticism from the Eysenck Personality Questionnaire (EPQ) or the Five Factor Model (FFM) inventory (see Glossary). Neuroticism is a higher order trait that measures a predisposition to experience anger, anxiety, depression, guilt, and other negative emotions or cognitions. A significant relationship between neuroticism and suicidal ideation has been regularly demonstrated [ 11, 22, 40, 41], and the same holds true for neuroticism and attempted suicide [ 28, 42–46]. High levels of anxiety or negative affectivity have been equally implicated in completed suicide [ 47–50].

A number of studies have reported introversion to be associated with suicidal thoughts and behaviour [ 42, 51–53]. In two psychological autopsies of completed suicides, Duberstein et al. [ 50] detected lower levels of extraversion in their cohort, and Maser et al. [ 53] found that people who committed suicide were shyer and less optimistic than other affectively ill patients. Extroversion has been shown to predict levels of social support [ 54, 55], perhaps explaining the association between introversion and suicide.

## Are the Suicide-Associated Personality Traits State-Independent?

Suicide attempts may occur in the context of an acute psychiatric crisis, biasing self-report measures of personality. One way of mitigating this problem is to use longitudinal data. In a study of 921 three-year-old children, Caspi et al. [ 52] found that impulsive toddlers were at an increased risk for suicide attempts at age 21. Fergusson et al. [ 11] followed a birth cohort for 21 years and similarly reported that high neuroticism and novelty-seeking were risk factors for suicidal behaviour. Similarly, a prospective study of affectively ill patients by Maser et al. [ 53] identified impulsivity as a key risk factor for both completed and attempted suicide, and Epstein et al. [ 48] found an association between high impulsivity and completed suicide.

Epstein et al. [ 48] also found that physicians who committed suicide had shown increased levels of hostility. Berglund [ 56] detected more irritability or aggression in people with alcoholic dependence who completed suicide. Among children who received treatment at a child guidance clinic, aggressive feelings and acts were predictive of later suicide [ 57], while among adult men both spontaneous and reactive aggression scores were elevated in later suicides [ 58].

While depressed affect certainly has the potential to bias personality data, there is enough evidence from longitudinal studies to support the hypothesis that certain personality traits, in particular hostility–aggression and impulsiveness, predispose to suicidal acts. In the next section, we examine the genetic basis of some of these personality traits.

## Is Personality Heritable?

As with the diathesis for suicide, personality or temperament is known to be at least partly heritable. The two most widely used personality models in genetic investigations of personality are the FFM [ 59] and Cloninger and colleagues' Biopsychosocial Model [ 60]. Twin studies have suggested heritability estimates for the FFM personality traits of between 40% and 60% [ 61–63], and comparable results have been obtained for Cloninger's Temperament and Character Inventory's (TCI) [ 60].

Impulsivity and aggression, traits which are slightly peripheral to the “traditional” dimensions of personality described by the FFM and the TCI, also display significant heritability values. These have been estimated to be approximately 40% [ 64, 65], 47% [ 66], 72% [ 67], and 80% [ 68]. Molecular genetic studies have lent support to the notion that personality is at least partially heritable.

Functional variants of the mono-amine oxidase A (MAO-A) and catechol-O-methyl-transferase (COMT) genes have been associated with violence and aggression [ 69–72], while Lesch et al. [ 73] found evidence indicating that the short allele of a functional insertion–deletion variant in the SERT gene was associated with anxiety-related personality traits. A plethora of replication attempts have met with mixed success, although a recent review suggested that the association is genuine [ 74].

## Do Personality and Suicidality Co-Segregate in Families?

This question is immediately problematic to answer because not one but many personality characteristics have been implicated in suicidality. Should an individual who has engaged in suicidal acts show increased anxiety, hostility–aggression, impulsiveness, and introversion, some combination of these traits or just one of these characteristics? How many different personality traits are enough to indicate a predisposition to suicidal behaviour? At this time, no pertinent evidence exists for this question to be accurately answered.

## Are Particular Personality Traits Found in Unaffected Family Members More Frequently than in the General Population?

To the best of our knowledge only two papers have touched on this issue. Brent et al. [ 75] found that the offspring of siblings concordant for suicidal behaviour displayed higher levels of impulsive aggression than the offspring of siblings discordant for suicide attempts and an unrelated control group. Kim et al. [ 76] reported an increased prevalence of aggression—defined as verbal or physical aggression towards others—in the first-degree relatives of suicide completers compared with a control group.

## Personality as an Endophenotype for Suicidality: An Evaluation

The same functional polymorphisms of SERT, MAO-A, and COMT that have been implicated in personality traits such as negative affectivity, aggression, and impulsiveness have been statistically associated with suicide or suicide attempts. This association raises the possibility that the relevant genetic variants contribute to the risk of suicide via their effect on personality.

Nevertheless, the endophenotypic approach to gene identification has perhaps been too easily accepted as a panacea for the ills of modern genetics. Some critical questions need to be raised.

First, does the status of SERT, MAO-A, COMT, and TPH as prime candidates for involvement in suicide owe anything to their putative role in anxiety-related, hostile, or impulsive behaviour? The answer is most probably no. The link between suicidal behaviour and abnormalities of the serotonergic system was recognised at least as far back as 1976 [ 77, 78]. Endophenotypes should facilitate the identification of candidate genes, but in this case it seems as though analyses of candidate genes are helping to identify endophenotypes.

Second, are anxiety-related, aggressive, and impulsive traits really genetically simpler than suicidal behaviour? Perhaps they are less complex in neurobiological terms but are they simple enough to significantly enhance success? For example, the potential for aggression may be an evolved trait that is characteristic of all mammalian species [ 79]. The key variable might therefore be the threshold at which aggression is elicited rather than self-reported aggression per se. The proximate environmental factors that elicit this behaviour and the historical childhood environment that potentially contributes to this threshold are thus variables that should not be ignored. The challenge of modelling these effects in genetic investigations is significant.

Third, can a consensus be reached on the optimum way of measuring traits such as impulsivity and aggression? In our review of the literature we encountered at least seven measures of hostility–aggression and four measures of impulsivity. The diversity of phenotypic assessment tools makes comparisons across studies difficult and raises the spectre of false positive results from multiple testing.

## Conclusion

A deeper understanding of the biology of suicidal behaviour may facilitate the development of new pharmacological interventions that could be targeted at vulnerable individuals, potentially saving thousands of lives. Identifying the genes that contribute to the risk for suicidal behaviour is an integral part of this process. While tantalising genetic clues are beginning to emerge from the mass of data, replication is a problem and new strategies such as endophenotyping are thus evolving.

In principle, the use of endophenotypes is an excellent strategy for parsing complex biological systems into their genetically simpler components and holds out much promise for bridging the chasm between research and clinical practice. The choice of the correct endophenotype is, however, of fundamental importance. Given the complexity of personality and the myriad ways in which it can be quantified, we have reservations about the fruitfulness of this approach. Future research should aim to identify a simpler endophenotype—an endophenotype of personality—that will introduce less noise into genetic analyses. One emerging possibility is the extensive body of work describing asymmetrical prefrontal cortical activation as evinced by electroencephalograph (EEG) and its relationship to positive (appetitive drive or behavioural activation) and negative (inhibitory) affective style; see [ 80, 81] for reviews. Other neuroimaging modalities such as functional magnetic resonance imaging (fMRI) may also facilitate the identification of the neural correlates of impulsive and aggressive dispositions.

Genetic knowledge is without peer in its potential for radically altering the practice of medicine, but the obstacles, especially in the field of psychiatric genetics, are formidable, and care should be taken not to overestimate the probability of short-term success. Advances are likely to be incremental but invaluable.

**Five Key Papers on Gene Studies and Suicide**

**Brent et al., 2003 [ 75].** The authors demonstrate that the familial loading for suicidal behaviour is mediated by impulsive–aggressive personality traits.

**Caspi et al., 1996 [ 52].** The authors showed that undercontrolled (impulsive) and inhibited three-year-olds were more likely than comparison children to have attempted suicide by the age of 21.

**Gottesman and Gould, 2003 [ 27].** A detailed discussion of the concept of the endophenotype, including its origins and its future use in psychiatry.

**Lesch et al., 1996 [ 73].** The first paper to reveal an association between a variant of the serotonin transporter gene and anxiety-related personality traits.

**Roy et al., 1991 [ 23].** One of the first studies to demonstrate that genetic factors impact suicidal behaviour.

**Glossary**

**Eysenck Personality Questionnaire** (EPQ): Measures three different dimensions hypothesised to underpin personality—neuroticism, psychoticism, and extraversion [ 82].

**Five Factor Model** (FFM): Reduces personality to variation along five dimensions— extraversion, neuroticism, conscientiousness, agreeableness, and openness to experience [ 59].

**Biopsychosocial Model:** Cloninger's model of personality [ 60] that differentiates between temperament factors which have a greater genetic loading, and character traits which are heavily influenced by the environment.

**Temperament and Character Inventory** (TCI): The questionnaire used to measure the seven dimensions of personality proposed in the Biopsychosocial model. The four temperaments are harm avoidance, novelty seeking, reward dependence, and persistence. Character traits are self-directedness, cooperativeness, and self-transcendence [ 60].

## Abbreviations

COMT: catechol-O-methyl-transferase
EPQ: Eysenck Personality Questionnaire
FFM: Five-Factor Model inventory
MAO-A: monoamine oxidase A
SERT: serotonin transporter
TCI: Cloninger's Temperament and Character Inventory
TPH: tryptophan hydroxylase
